# EF-hand protein, EfhP, specifically binds Ca^2+^ and mediates Ca^2+^ regulation of virulence in a human pathogen *Pseudomonas aeruginosa*

**DOI:** 10.1038/s41598-022-12584-9

**Published:** 2022-05-25

**Authors:** Biraj B. Kayastha, Aya Kubo, Jacob Burch-Konda, Rosalie L. Dohmen, Jacee L. McCoy, Rendi R. Rogers, Sergio Mares, Justin Bevere, Annalisa Huckaby, William Witt, Shuxia Peng, Bharat Chaudhary, Smita Mohanty, Mariette Barbier, Gabriel Cook, Junpeng Deng, Marianna A. Patrauchan

**Affiliations:** 1 Department of Microbiology and Molecular Genetics, Oklahoma State University, Stillwater, OK 74078 USA; 2 Department of Biochemistry and Molecular Biology, Oklahoma State University, Stillwater, OK 74078 USA; 3 Department of Chemistry, Oklahoma State University, Stillwater, OK 74078 USA; 4 Vaccine Development Center at West Virginia University, Morgantown, WV 26506 USA; 5 Department of Microbiology, Immunology and Cell Biology, West Virginia University, Morgantown, WV 26506 USA

**Keywords:** Biochemistry, Microbiology, Molecular biology

## Abstract

Calcium (Ca^2+^) is well known as a second messenger in eukaryotes, where Ca^2+^ signaling controls life-sustaining cellular processes. Although bacteria produce the components required for Ca^2+^ signaling, little is known about the mechanisms of bacterial Ca^2+^ signaling. Previously, we have identified a putative Ca^2+^-binding protein EfhP (PA4107) with two canonical EF-hand motifs and reported that EfhP mediates Ca^2+^ regulation of virulence factors production and infectivity in *Pseudomonas aeruginosa,* a human pathogen causing life-threatening infections. Here, we show that EfhP selectively binds Ca^2+^ with 13.7 µM affinity, and that mutations at the +X and −Z positions within each or both EF-hand motifs abolished Ca^2+^ binding. We also show that the hydrophobicity of EfhP increased in a Ca^2+^**-**dependent manner, however no such response was detected in the mutated proteins. ^15^ N-NMR showed Ca^2+^-dependent chemical shifts in EfhP confirming Ca^2+^-binding triggered structural rearrangements in the protein. Deletion of *efhP* impaired *P. aeruginosa* survival in macrophages and virulence in vivo*.* Disabling EfhP Ca^2+^ binding abolished Ca^2+^ induction of pyocyanin production in vitro. These data confirm that EfhP selectively binds Ca^2+^, which triggers its structural changes required for the Ca^2+^ regulation of *P. aeruginosa* virulence, thus establishing the role of EfhP as a Ca^2+^ sensor.

## Introduction

Calcium ions (Ca^2+^) have been recognized for their versatile signaling roles, building the core of cell communication network in eukaryotes. Ca^2+^ signaling regulates numerous eukaryotic processes, including host defenses against bacterial infections^1,2^. Abnormalities in Ca^2+^ cellular homeostasis may lead to human diseases^3^ or result from diseases, for example, bacterial infections^4–6^*.* The altered levels of extracellular Ca^2+^, as exemplified by elevated Ca^2+^ in nasal and lung liquids of Cystic Fibrosis (CF) patients^7^, may serve as host factors driving patho-evolution of invading pathogens. Therefore, studying Ca^2+^ signaling and regulation in human pathogens is of high importance.

*P. aeruginosa* is a human pathogen responsible for life threatening acute and chronic infections, including pneumonia, infective endocarditis and infections in urinary tract, skin, burn and surgical wounds^8–11^. It is also responsible for at least 11% of all nosocomial infections occurring in the United States^12^. *P. aeruginosa* is particularly well known for causing lethal lung infections in CF patients^13,14^. The persistence of the pathogen during infections is commonly attributed to its potent multifactorial virulence and abilities to adapt to the host environment and become resistant to antibiotics and host defenses^15^.

Our earlier studies showed that *P. aeruginosa* responds to elevated levels of extracellular Ca^2+^ through increased production of biofilm, secreted virulence factors such as pyocyanin, pyoverdine, and proteases^16^, enhanced virulence in plant^17^ and worm^18^ models, and increased resistance to antibiotics^19^. Two major Ca^2+^ regulatory pathways have been reported, including the Ca^2+^-induced two-component regulatory system CarSR^20^ and the membrane Ca^2+^ sensor LadS kinase which activates the Gac/Rsm system, controlling the switch to sessile mode of growth^21^. We proposed that Ca^2+^ response is mediated by intracellular Ca^2+^ signaling. The latter is supported by three main observations: *P. aeruginosa* tightly regulates the low resting cytoplasmic concentration of Ca^2+^ ([Ca^2+^_cyt_]) at µM level, the organism generates transient increases in [Ca^2+^_cyt_] in response to external Ca^2+^^17^, and the discovery of Ca^2+^ channel CalC that is required for generating the [Ca^2+^_cyt_] transient spikes controlling Ca^2+^-dependent regulation of gene expression (Guragain, et.al. in submission). However, the molecular mechanisms responsible for recognizing the fluctuations in Ca^2+^ levels and transducing Ca^2+^ signals towards cellular processes are not known.

We previously identified a putative EF-hand protein encoded by gene PA4107. Based on the presence of two EF-hand motifs known to selectively bind Ca^2+^^22^, the protein was named EfhP and predicted to bind Ca^2+^. We showed that EfhP plays an important role in Ca^2+^-regulated production of pyocyanin and alginate, resistance to oxidative stress and *P. aeruginosa* infectivity in plants^17^. EF-hand containing proteins belong to one of the largest family of proteins, some of which function as Ca^2+^ sensors, signal transducers, or modulators. One of the most well studied and versatile EF-hand Ca^2+^ sensors is calmodulin (CaM). CaM is known to bind Ca^2+^, which leads to conformational rearrangements that reveal its hydrophobic clefts and allow specific interactions with numerous binding partners. The altered activities of CaM protein targets define cellular responses to fluctuating Ca^2+^ (reviewed in^23^). Considering the presence of EF-hand motifs in EfhP amino acid sequence and its role in Ca^2+^-regulated processes in *P. aeruginosa*, we predicted that, similarly to CaM, EfhP binds Ca^2+^, which triggers its conformational changes and enables signal relaying towards the downstream responses. To test this hypothesis, we expressed the recombinant EfhP from *E. coli* and characterized its binding with Ca^2+^ and the triggered structural changes by a combination of isothermal titration calorimetry (ITC), fluorospectrophotometry, and nuclear magnetic resonance (NMR) approaches. Through site-directed mutagenesis, we identified the amino acid residues in EfhP that are key to Ca^2+^ binding*.* Finally, we studied the roles of EfhP in the Ca^2+^-regulated production of virulence factor pyocyanin in *P. aeruginosa *in vitro and virulence of the pathogen in animal model in vivo. The results of this study establish EfhP as a Ca^2+^ sensor and provide insights into better understanding of Ca^2+^ signaling in bacteria.

## Results

### EfhP is highly conserved in *Pseudomonas aeruginosa*

Sequence analysis of EfhP (PA4107, *pseudomonas.com*) revealed two canonical EF-hand motifs forming a conserved pattern DxDxDG with identical key residues in the positions 1(+X), 3 (+Y), 5(+Y), 9(−X), and 12 (−Z) (Table 1, Fig. S1A) known to coordinate Ca^2+^ (reviewed in^22,24^). Another residue shared by both EfhP EF-hands is glycine (G) at the position 6. This residue is highly-conserved among EF-hand proteins and known for enabling the loops to encompass Ca^2+^, which is critical for the high binding affinity^25^. Modeling the 3D structure of EfhP by I-TASSER using CaM as the top threading model (Z score of 1.94) supported the prediction of two canonical helix-loop-helix structures for EF-hand motifs (Fig. S2A). To evaluate the conservation of EfhP among bacteria, we performed amino acid sequence alignments using BLASTP and non-redundant NCBI database. At the time of the analysis, we revealed that almost 800 full-length protein homologs of EfhP sharing at least 25% sequence identity are present in 45 bacterial genera. The majority of the hits (65%) belong to *Pseudomonas*. These pseudomonads’ homologs share up to 100% identity, whereas homologs in other genera, including *Stenotrophomonas* with the second highest number of EfhP homologs (12%)*,* share up to 58% sequence identity. When searching for homologs among all sequenced genomes of *Pseudomonas* species using the pseudomonas genome database (www.pseudomonas.com), we found that 3040 genomes carry homologs of EfhP with above 42.6% amino acid sequence identities (calculated over the full length of EfhP). Interestingly, 98.5% of these homologs were present in *P. aeruginosa.* We took a closer look at the remaining 1.5%, most of which belong to *Pseudomonas sp.,* and performed the average nucleotide identity (ANI) analysis to retrieve their species identity. Based on the ANI, all the 26 *P. sp.* strains and three single strains of *P. mendocina*, *P. otitidis and P. protegens* were identified as *P. aeruginosa* (Fig. S2B). These data suggest that the full-length EfhP is conserved predominantly in *P. aeruginosa*.

**Table 1:** Primary sequences of EF-hand Ca^2+^-binding loops from EfhP and CaM.

Protein	Ca^2+^-binding loop	+X 1	2	+Y 3	4	+Z 5	6	−Y 7	8	−X 9	10	11	−Z 12
EfhP	I	*D*	T	*D*	**H**	*D*	*G*	K	V	*S*	R	*A*	*E99*
	II	*D*	S	*D*	**H**	*D*	*G*	F	I	*S*	E	*A*	*E126*
CaM	I	*D*	K	*D*	G	*D*	*G*	T	I	T	T	K	*E31*
	II	*D*	A	*D*	G	N	*G*	T	I	D	F	P	*E67*
	III	*D*	K	*D*	G	N	*G*	Y	I	*S*	A	*A*	*E104*
	IV	*D*	L	*D*	G	*D*	*G*	G	V	N	Y	E	*E140*

Ca^2+^-binding sites are numbered, Ca^2+^-coordinating residues are labeled as sites X, Y, Z, −Y, −X, and −Z. Identical residues in EfhP loops are shown in bold, identical residues in EfhP and CaM are in italics. The 12th glutamate residues are shown with the coordinates in the corresponding proteins.

**Figure 1:**
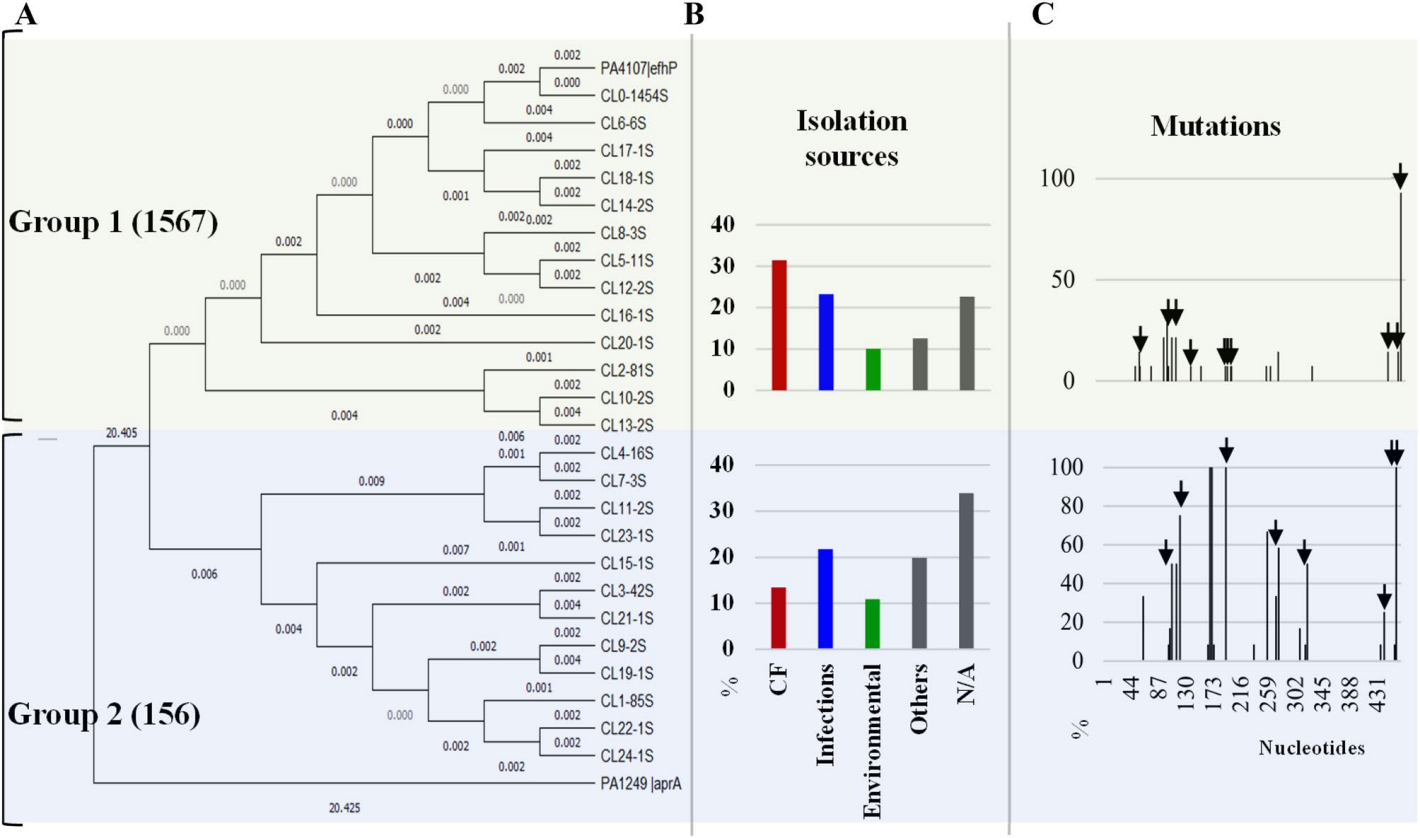
Correlative analyses of phylogenetic relationship, isolation sources, and frequency of mutations in *efhP* homologs. (**A**) The 1723 sequences of *efhP* homologs and *efhP* were clustered using CD-Suite^26^ with a cutoff at 0.9995. One representative from each cluster was used to build a phylogenetic tree in MEGA^27^. The branch lengths show the evolutionary time between two nodes. PA1249, which encodes an EF-hand containing protease, AprA, was included as an outgroup. (**B**) Clustering sequence similarities with the isolation sources formed two apparent groups divided by the horizontal line. Top group represents 1567 sequences, and the bottom group represents 156 sequences. The isolation sources shown are grouped into main categories that represent at least 60% of the sequences shown in (Fig. S2C). All other sources are represented as ‘Others’. (**C**) Mutation profiles are depicted for each group. The % of *efhP* homologs carrying the depicted mutations are plotted. The arrows indicate missense mutations.

Considering that *P. aeruginosa* is known to reside in a variety of ecological niches, we hypothesized that conservation of *efhP* sequence reflects niche-specific adaptations and therefore correlates with the isolation source of the gene-carrying strains. To test this, we retrieved all the available isolation sources for strains carrying full-length *efhP* homologs and applied multiple sequence analyses followed by clustering with the isolation sources. The analyses revealed that 86.7% of the retrieved homologs belong to strains isolated from clinical sources (clinical isolates from patients, hospitals, etc.) and the other 13.2%—from environmental (soil, natural water reservoirs, etc.). Among those representing clinical sources, a majority came from CF clinical samples (Fig. S2C). This, however, likely reflects the distribution of sequencing efforts. To evaluate potential correlation between sequence variations among *efhP* homologs and the isolation sources, we clustered 1723 sequences using CD-HIT suite at 99.5% similarity cutoff, aligned cluster representatives using ClustalW in MEGA, and used the alignment to construct maximum likelihood phylogenetic trees in MEGA with PA1249, encoding EF-hand containing protease AprA, as an out-group (Fig. 1A). Considering the phylogenetic relationship and the isolation sources, the clusters fell into two groups. Interestingly, 31.5% of sequences in the first group originated from CF isolates, compared to 13.5% of the sequences in the second (Fig. 1B). Although the overall distribution of the mutations within the sequence were similar in the two groups, a smaller number of mutations was identified in the first group that increased significantly in the second (Fig. 1C). Considering that CF isolates represent the largest number of sequences (31.5%) in the largest top group (n = 1567), these data indicate a higher conservation of *efhP* homologs among CF isolates. The functional significance of these mutations will be the focus of further studies.

To further investigate the conservation of EfhP in *P. aeruginosa,* we calculated the percentage of EfhP-harboring *P. aeruginosa* strains among different groups of isolates (Fig. S3A). We determined that 64% of all the complete and partially sequenced *P. aeruginosa* genomes (4643 at the time of the analyses) encode for EfhP. These include 79% of CF isolates, 59% of non-CF clinical isolates, and 62% of environmental isolates and show a greater incidence of EfhP among CF isolates. We also plotted the distribution of amino acid sequence identities between EfhP and its full-length homologs in these groups of isolates (Fig. S3B). The highest number of EfhP homologs (351) from CF strains share the highest percent identity (99.6–100), followed by non-CF clinical isolates (300) and environmental (77). These observations further support a higher conservation of EfhP among CF isolates.

### EfhP plays an important role during infection

**Figure 2:**
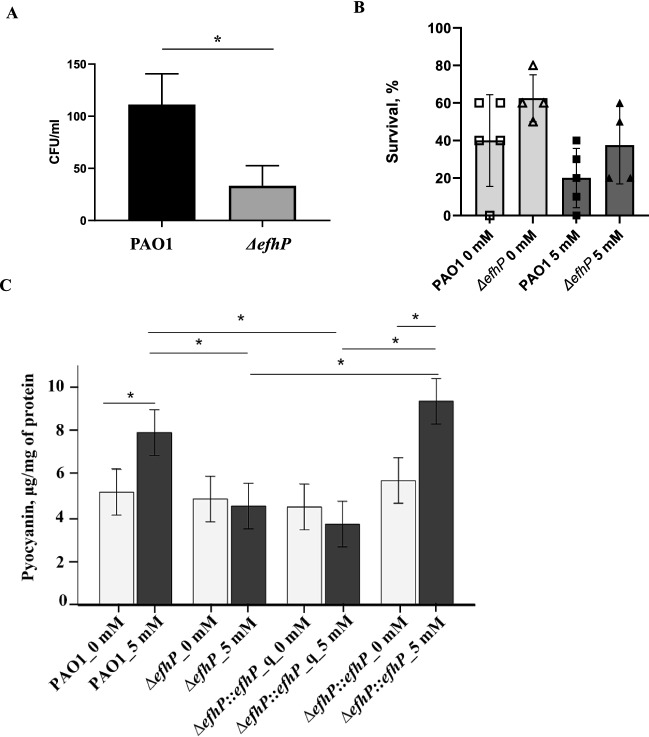
The role of *efhP* in *P. aeruginosa* PAO1 virulence. (**A**) Survival of *P. aeruginosa* PAO1 and Δ*efhP* in J774A.1 murine macrophages. Intracellular survival was measured 90 min after infection by using a gentamycin exclusion assay. Each number corresponds to the average of three technical replicates, each experiment was repeated three times on three different days. (*) Student’s T-test *p* < 0.05 (**B**) Percent survival of *G. mellonella* injected with 2–5 CFU of PAO1 and Δ*efhP* grown at no added CaCl_2_ (empty symbols) or at 5 mM CaCl_2_ (filled symbols). Cells were grown in BMM with the corresponding level of CaCl_2_. Every data point represents an individual experiment. Each experiment used 10 experimental and five control animals. (*) One-way ANOVA *p* < 0.05 (**C)** Pyocyanin production during growth on agar surface of PAO1, Δ*efhP,* Δ*efhP::efhP_q* and Δ*efhP::efhP*. (*) Univariate ANOVA *p* < 0.05.

Previously, we showed that EfhP contributes to the ability of *P. aeruginosa* to infect and cause disease in lettuce leaves^17^. We hypothesized that the protein plays a similar role in *P. aeruginosa* virulence in macrophage and animal models of infection. To test this hypothesis, we used murine macrophages and *G. mellonella* infection models. J774A.1 murine macrophages were infected with wild type PAO1 and Δ*efhP*, and intracellular survival was measured after 90 min by using a gentamycin exclusion assay. The deletion of *efhP* significantly reduced the survival in comparison to the wild type (Fig. 2A). We also tested the survival of *G. mellonella* larvae after infecting with PAO1 and Δ*efhP.* This model enabled injecting bacteria at no or 5 mM added CaCl_2_. *G. mellonella* infected with PAO1 grown and injected at no added Ca^2+^ reached, on average, 40% survival (Fig. 2B). In contrast, the larvae injected with Δ*efhP* mutant grown under these conditions showed 62.5% survival. In the presence of 5 mM CaCl_2_, the survival of the larvae infected by PAO1 reduced by two-fold, and the larvae infected with the mutant showed about 25% reduction in survival (Fig. 2B). These data showed that *efhP* deletion leads to a 1.5-fold decrease of the virulence in the cells grown at no Ca^2+^ and 1.8-fold decrease in the cells grown at 5 mM Ca^2+^. Together, these data indicate that *efhP* contributes to the regulation of *P. aeruginosa* survival and virulence.

### The structure of EfhP is stabilized by Ca^2+^ binding

**Figure 3:**
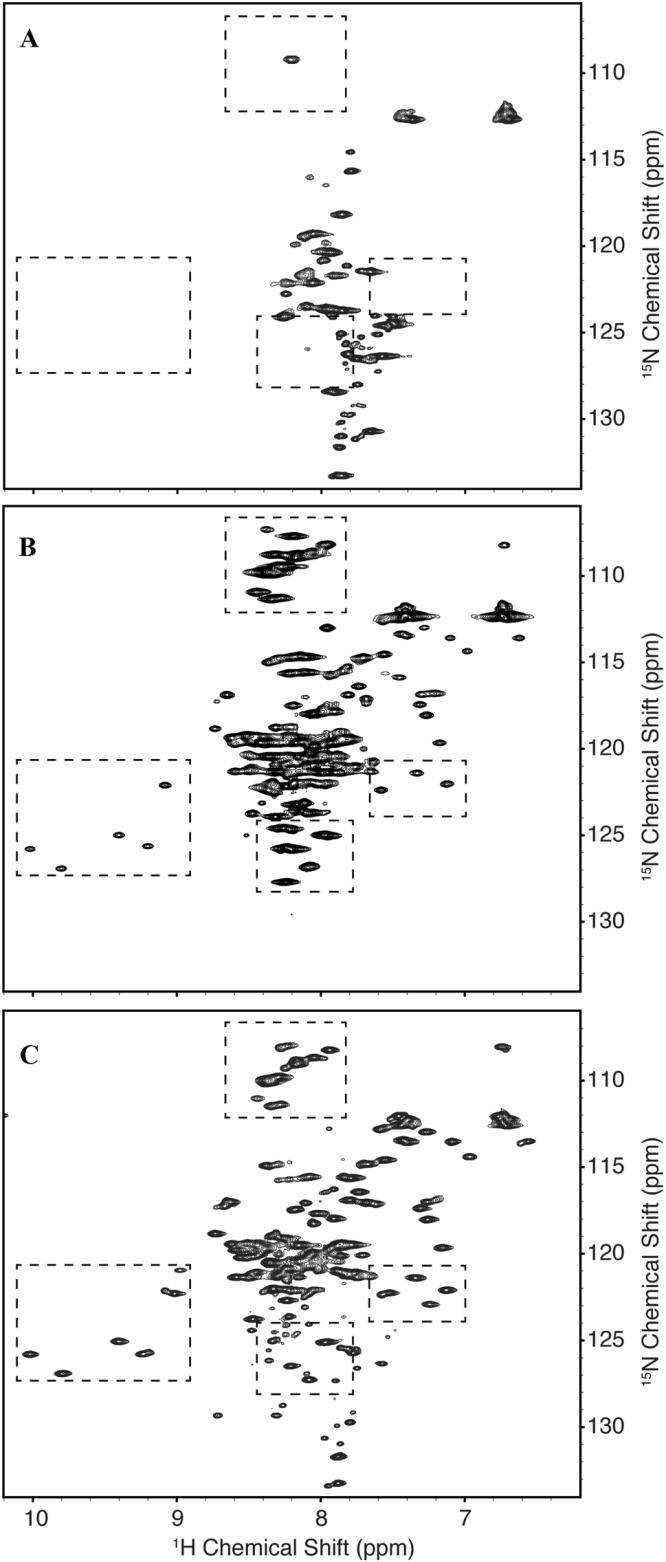
NMR analyses of EfhP binding Ca^2+^. ^1^H-^15^ N HSQC spectra of EfhP_tr_, (**A**) decalcified, (**B**) non-decalcified, and (**C**) non-decalcified with the addition of 1.5 mM CaCl_2_. Regions of significant changes in the spectra are highlighted with dashed boxes.

To characterize the EfhP-Ca^2+^ interactions, the protein lacking the signal peptide (EfhP_tr_) was expressed in *E. coli* and purified (as described in the Methods). EfhP_tr_ contains 123 amino acids (33 to 155) with a calculated molecular weight of 12.9 kDa (Fig. S4C). Circular Dichroism (CD) and NMR spectroscopy were used to study the structural changes in EfhP_tr_ in the presence of Ca^2+^. The CD spectra of EfhP_tr_ prior to decalcification showed minima near 210 and 222 nm, indicative of helical content (Fig. S4A). Incremental addition of Ca^2+^ lead to a slight increase in ellipticity suggesting slight conformational transitions in response to interactions with the ion. When the decalcified protein was titrated with Ca^2+^, the CD spectra showed a combination of noise around 200–208 nm and about tenfold increase in the ellipticity at no Ca^2+^ followed by Ca^2+^-dependent decrease in the ellipticity for the 210 and 220 nm minima (Fig. S4B). These observations suggest that EfhP_tr_ binds Ca^2+^ and that freeing the protein from the ion destabilizes its structure. In agreement, the ^1^H-^15^ N-HSQC spectra of Ca^2+^-free EfhP_tr_ showed poorly resolved amide resonances and a narrow dispersion of signals with ^1^H chemical shifts clustered between 7.4 and 8.4 ppm (Fig. 3A), also suggesting that decalcifying the protein prevents the formation of a stable, folded structure and likely leads to a molten globule state^28^. A similar behavior was observed for a number of other EF-hand proteins^29,30^. However, the spectra of non-decalcified EfhP_tr_ were well-resolved and notably more disperse, displaying backbone resonances with chemical shifts ranging from 6.6 to 10.0 (Fig. 3B). The number of visible peaks in these spectra were consistent with the number of residues in the protein, a sign that the structure of the protein in these samples was stabilized. The incremental addition of CaCl_2_ up to 1.5 mM (5:1, calcium to protein) led to significant changes in the ^1^H-^15^ N heteronuclear single-quantum correlation (HSQC) spectrum of the protein, indicating structural rearrangements upon Ca^2+^ binding (Fig. 3C). The observed downfield-shifted peaks corresponded to those that are characteristic of the globular core of the EF-hand Ca^2+^-binding domain^31^. In addition to chemical shift changes, several new peaks became visible in response to Ca^2+^ addition, suggesting a stabilization of a separate conformation from the holo structure. Like the non-decalcified spectrum, the calcium supplemented samples displayed a broader dispersion of signals from 6.6 and 10.0 ppm in the proton dimension but had an expanded dispersion ranging from 108 to 133 ppm in the nitrogen dimension. The areas with the most significant changes are outlined in the Fig. 3C. Overall, the CD and NMR data agree and demonstrate that EfhP requires Ca^2+^ for its stability and that the protein undergoes structural rearrangements upon binding the ion.

### EfhP selectively binds Ca^2+^

**Figure 4:**
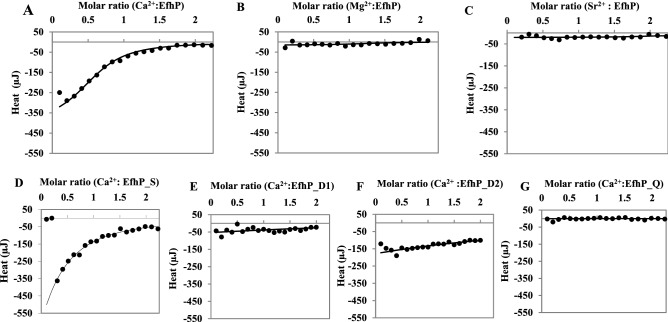
Isothermal Titration Calorimetry analysis of EfhP_tr_ and mutant proteins. Binding curves fitted to the model of a single set of binding sites of 100 µM EfhP titrated with 1 mM Ca^2+^ (**A**), Mg^2+^ (**B**), or Sr^2+^ (**C**); and EfhP_S (**D**), EfhP_D1 (**E**), EfhP_D2 (**F**) and EfhP_Q (**G**) titrated with 1 mM of Ca^2+^_._ The proteins and ligands were prepared in 20 mM HEPES buffer with 100 mM NaCl, pH 7.8. HEPES buffer titrated with the corresponding ligand was subtracted as the blank for each titration.

To further study Ca^2+^-binding by EfhP, we employed Isothermal Titration Calorimetry (ITC). The ITC experiments revealed that titrating EfhP_tr_ with Ca^2+^ (Table 2, Figs. 4A, S5, S6) leads to exothermic binding with enthalpy (ΔH) of − 40.2 kJ/mol. For understanding the interactions between EfhP_tr_ and Ca^2+^, we considered three modes of modeling (Microcal PEAQ-ITC Analysis Software 0.9.0.1252). The model of sequential binding fit poorly, whereas the models of one-set of binding sites and two-sets of binding sites resulted in similar fits with a slightly better statistics for the former (data not shown). Thus, we assumed the one-set model and calculated the dissociation constant, Kd of 13.7 µM (Table 2). The number of binding sites (N) was predicted to be about 1, indicating that only one EF-hand binds Ca^2+^. To eliminate the possibility of the residual Ca^2+^ preoccupying one EF-hand, we decalcified and then titrated EfhP_tr_ with Ca^2+^. This did not significantly alter the Kd (13.2 µM, Table 2) of the binding but predicted an N value of about 2. This suggests that one EF-hand site was preoccupied with Ca^2+^ and cleared during the decalcification procedure, supporting the expectation that each EF-hand binds one Ca^2+^ ion. In contrast, when Mg^2+^ or Sr^2+^ were used for titration (Figs. 4B, C, S5), almost no heat was generated, indicating that EfhP does not bind Mg^2+^ nor Sr^2+^. These results demonstrate that EfhP preferentially binds Ca^2+^, which supports the regulatory role of Ca^2+^ binding and the potential function of EfhP as a Ca^2+^ sensor.

**Table 2:** Thermodynamic parameters for the binding of Ca^2+^ to EfhP and its mutated variants.

Protein	N	Kd (µM)	∆H (kJ/mol)
EfhP	0.6	13.7	− 40.2
EfhP_decalcified	1.8 ± 0.5	13.2 ± 0.4	− 54.7 ± 18.8
EfhP_S	0.2	77.2	− 271.4
EfhP_D1	No binding	N/A	N/A
EfhP_D1_decalcified	No binding	N/A	N/A
EfhP_D2	No binding	N/A	N/A
EfhP_D2 decalcified	No binding	N/A	N/A
EfhP_Q	No binding	N/A	N/A

The proteins were decalcified by using Chelex-100 resin column. The residual Ca^2+^ measured by BAPTA assay was equal or below 0.8 μM. The ITC data were analyzed by using One-Set of Sites binding model.

To investigate the influence of the individual EF-hands on Ca^2+^ binding, we generated several point mutations by replacing the amino acids at positions +X or/and −Z in the first, the second or both EF-hand motifs in the EfhP_tr_ (Fig. S1). The single mutant EfhP_S (D88N), double mutants EfhP_D1 (D88N/E99Q) and EfhP_D2 (D115N/E126Q), as well as the quadruple mutant EfhP_Q (D88N/E99Q/D115N/E126Q) were subjected to Ca^2+^ titration and ITC. The titration of the single mutant.

EfhP_S showed a sixfold increase in the Kd (77.2 µM) in comparison to that of the wild type EfhP_tr_ (Table 2, Figs. 4D, S5D). The titration of EfhP_D1, EfhP_D2, and EfhP_Q by Ca^2+^ showed no Ca^2+^ binding (Table 2, Figs. 4E,F,G, S5, S6). These data indicate that mutating D1 of the 1st EF hand reduced the Ca^2+^ binding in EfhP, however replacing both D1 and E12 of each or both EF-hand motifs completely abolished the protein’s ability to bind Ca^2+^.

### Binding Ca^2+^ increased EfhP hydrophobicity

**Figure 5:**
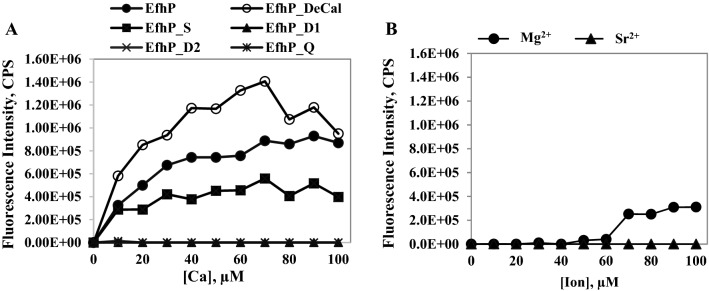
Fluorospectrophotometry analyses of EfhP_tr_ and mutant proteins. (**A**) The changes in fluorescence intensity of 1,8-anilinonaphthalene sulfonate (ANS) interacting with EfhP, decalcified EfhP (EfhP_DeCal), single (EfhP_S) double (EfhP_D1 and EfhP_D2) and quadruple EfhP (EfhP_Q) mutant proteins titrated with Ca^2+^. (**B**) The changes in ANS fluorescence intensity of EfhP titrated with Mg^2+^ or Sr^2+^. EfhP and the mutant proteins (10 µM) were mixed with 50 µM of ANS and titrated with 1 M Ca^2+^, Mg^2+^ or Sr^2+^. A total of ten 10 µl aliquots of these ligand solutions were added to achieve the final concentrations of 10 to 100 µM. The emission spectra were acquired at excitation of 350 nm. The fluorescence values of the buffer alone and protein-ANS mixture were subtracted as a blank. Each titration was repeated three times. The fluorescence intensity was detected in counts per second (CPS).

Based on the overall resemblance between EfhP and CaM, we predicted that Ca^2+^-binding would lead to EfhP conformational changes that in turn increase its hydrophobicity. To evaluate the changes in EfhP hydrophobicity in response to binding Ca^2+^, we used the hydrophobic fluorophore ANS, known to yield increased fluorescence upon interacting with hydrophobic molecules^32^. The fluorescence of EfhP_tr_-ANS complex was measured upon titrating with Ca^2+^, Mg^2+^, and Sr^2+^. A concentration-dependent increase in the fluorescence of EfhP_tr_-ANS complex was detected with increasing concentrations of Ca^2+^ up to 70 µM (Fig. 5A). To test a Ca^2+^-free protein, we decalcified EfhP_tr_ and titrated the EfhP_tr_-ANS complex with Ca^2+^. This further increased the fluorescence of the complex upon Ca^2+^ binding (Fig. 5A), indicating that when Ca^2+^-free EfhP binds Ca^2+^, it becomes even more hydrophobic. The increased hydrophobicity likely reflects Ca^2+^-dependent structural rearrangements in EfhP. In contrast, no significant increase in the ANS fluorescence was detected upon titration with Mg^2+^ or Sr^2+^ (Fig. 5B), which is consistent with the ITC data, demonstrating that EfhP does not bind these ions. The single mutant EfhP_S showed an increase in the fluorescence upon the addition of Ca^2+^, but the response quickly plateaued, and no significant change was observed after the third injection (Fig. 5A). In agreement with the ITC data, ANS complexes with EfhP_D1, EfhP_D2, and EfhP_Q mutant proteins showed no fluorescence increase in response to Ca^2+^ titration (Fig. 5A).

### Ca^2+^ binding is required for EfhP function

Previously, we showed that EfhP is required for production of virulence factor pyocyanin in the presence of elevated Ca^2+^ in alginate-overproducing strain of *P. aeruginosa* strain FRD1^17^. Here we hypothesized that EfhP plays role in Ca^2+^ regulation of pyocyanin production in the non-mucoid strain PAO1 as well, and that its ability to bind Ca^2+^ is required for this role. To test this hypothesis, we measured pyocyanin production in PAO1, deletion mutant (Δ*efhP*)*,* and two genetic complements with either wild type *efhP* (ΔefhP::*efhP*) or quadruple mutant *efhP* whose product was not able to bind Ca^2+^ (Δ*efhP*::*efhP_q*)*.* Pyocyanin was extracted from the cultures grown for 24 h on BMM plates with and without 5 mM Ca^2+^ (Fig. 2C). When grown without Ca^2+^, PAO1 produced 5.2 µg/mg pyocyanin normalized by total protein. The addition of 5 mM Ca^2+^ resulted in a 1.5 fold increase in pyocyanin production. In contrast, this induction was not observed in the Δ*efhP* and Δ*efhP*::*efhP_*q strains, for which the levels of pyocyanin remained unchanged upon Ca^2+^ addition (Fig. 2C). The complementation of Δ*efhP* with a wild type *efhP* recovered Ca^2+^-enhanced production of pyocyanin observed in PAO1. This indicates that deletion of *efhP* or abolishing the protein’s ability to bind Ca^2+^ results in the loss of Ca^2+^-induction of pyocyanin biosynthesis.

## Discussion

According to our previous studies^16^ and studies by others^33–35^, Ca^2+^ regulates important features of *P. aeruginosa* physiology and virulence. Considering this along with the importance of the ion as a host factor, identifying Ca^2+^ sensors and deciphering Ca^2+^ regulatory pathways is imperative for a better understanding of the mechanisms controlling *P. aeruginosa’s* virulence and for the development of novel strategies to prevent devastating infections caused by this pathogen. Here we show the first experimental evidence that EfhP (PA4107) possesses the key features of a Ca^2+^ sensor: it specifically binds Ca^2+^, undergoes Ca^2+^-dependent structural rearrangements which increase its hydrophobicity, and it transduces the Ca^2+^ signal towards cellular processes. The latter is illustrated by the observation that EfhP mediates Ca^2+^ regulation of virulence factor pyocyanin production in vitro*,* which requires its ability to bind Ca^2+^. We also show that EfhP plays an important role in *P. aeruginosa* survival and virulence in invertebrate animal model in vivo*.*

Bioinformatic analyses suggested that full-length EfhP is mostly prevalent among *Pseudomonas aeruginosa* strains. This opens an intriguing question about the evolutionary pressure that secures this protein in the genus. To gain additional insights, we related the sequence similarity between *efhP* homologs with the sources of the strains’ isolation. The analyses suggested that fewer mutations occur within the homologs encoded in CF isolates, and more frequent mutations correlate with environmental isolates. Although the results may be skewed due to disproportionate numbers of sequences available for clinical and environmental isolates, the observation supports the role of the protein in *P. aeruginosa* virulence. The higher incidence and conservation of EfhP among CF isolates likely reflects pathoadaptation of the bacterium as an evolutionary driver of EfhP conservation. The fact that *P. aeruginosa* genome contains no homologs of EfhP signifies the role of this protein and possibly contributes to its evolutionary conservation.

In order for a protein to serve as a Ca^2+^ sensor, it must first selectively bind Ca^2+^ ion(s). ITC showed that EfhP binds Ca^2+^ with a Kd of 13.7 µM. This value lies within the Kd values of other EF hand Ca^2+^ sensors varying in the nM to µM range^36^. Despite some similarities between Ca^2+^ and Mg^2+^ ions and the ability of some EF-hand motifs to bind Mg^2+^, the interactions between EF-hand proteins and these ions differ and lead to different functional consequences^37^. Based on their interactions with Mg^2+^, EF-hand proteins can be grouped into Ca^2+^-specific and Ca^2+^/Mg^2+^ -binding. According to the ITC data, EfhP showed no binding to Mg^2+^ (and Sr^2+^), likely due to stereochemical constraints (reviewed in^37^), indicating it belongs to Ca^2+^-specific EF-hand proteins. Further, according to ITC and ANS fluorescence spectroscopy, replacing the D residue at position +X in the first EF-hand motif significantly impairs the capability of EfhP for binding Ca^2+^. This EF-hand residue is highly conserved among EF-hand proteins^38^ and its replacement was shown to inhibit Ca^2+^ binding in CaM^39^. When both D and E residues at positions +X and −Z were replaced within each or both EF-hands, no Ca^2+^ binding was observed. The E12 (-Z) is also highly conserved and supplies two of the seven coordinating oxygens within the EF hand Ca^2+^-binding loop^24,40,41^. Overall, our data support the importance of D1(+X) and E12(−Z) residues for Ca^2+^ binding of EfhP and suggest that both EF-hands are needed to enable EfhP binding the ion. This indicates cooperative Ca^2+^ binding. Positive cooperativity is a key functional attribute of EF-hand proteins and is particularly important for Ca^2+^ sensors, where it provides the ability to detect the small changes in Ca^2+^ concentrations constituting Ca^2+^ signals^42,43^.

The second requirement for a functional Ca^2+^ sensor is the ability to transduce Ca^2+^ signals most commonly via Ca^2+^-triggered conformational changes which enable the selective binding of protein partners. This phenomena is well exemplified by CaM, which undergoes major conformational transitions upon binding Ca^2+^^44,45^ but not other ions^46^. These conformational changes expose hydrophobic surfaces of CaM and enable selective binding to a large number of diverse protein targets^47,48^. In contrast, EF-hand proteins that serve as Ca^2+^ buffers, for example, calbindin, bind Ca^2+^ without significant changes in their conformation^49,50^. Here we applied NMR spectroscopy and ANS-based fluorospectrophotometry which showed that the addition of Ca^2+^ triggers a significant change in the EfhP structure and hydrophobicity, respectively. The NMR-detected chemical shifts clearly illustrated binding Ca^2+^, which likely lead to not only local but also global rearrangements in the protein structure. Future research will focus on determining the residues affected by Ca^2+^ binding and building three-dimensional models of both the unbound and bound forms of EfhP, so that the more detailed mechanisms of binding can be determined. Overall, the observed Ca^2+^-dependent structural rearrangements in EfhP and the Ca^2+^-dependent increase in its hydrophobicity strongly support the ability of the protein to recognize and transduce Ca^2+^ signals.

According to the updated SignalP 5.0 prediction of the signal peptide (in contrast to the reported earlier transmembrane domain^51^), EfhP likely resides in the periplasm. Based on the *E. coli* study^52^, the periplasmic concentration of Ca^2+^ reflects or exceeds its extracellular levels. Considering the micromolar Kd for Ca^2+^, we predict that EfhP plays a particularly significant role in Ca^2+^ signaling in *P. aeruginosa* when the organism resides in the environments with micromolar levels of Ca^2+^. Examples of such environments include those within the host mucus or within *P. aeruginosa* biofilms. The low levels of Ca^2+^ in these environments are due to the presence of negatively charged polymers, such as F-actin, DNA, and alginate^53–55^, that chelate Ca^2+^ ions. These environments are clinically relevant and prevalent within the lungs of CF patients, where the mucus levels are particularly high^56^ and alginate-overproducing *P. aeruginosa* strains are predominant^57^. This prediction is supported by the previously observed more pronounced role of EfhP in the virulence of a mucoid FRD1 strain of *P. aeruginosa*^51^. Another example of low Ca^2+^ environment is within macrophages and neutrophils, which play an essential role in host defense against bacterial pathogens. The level of phagosomal Ca^2+^ decreases during phagocytosis from the low millimolar extracellular level to low micromolar level^58^. Here we show that EfhP plays role in PAO1 survival during phagocytosis, further supporting the idea of the protein’s importance for Ca^2+^ regulation at micromolar levels of the ion.

In bacteria, a number of EF hand-proteins have been reported (reviewed in^59^) and a few have been proposed to function as Ca^2+^ sensors (reviewed in^60^). Although some of these proteins were shown to play important roles in virulence and host–pathogen interactions, their Ca^2+^ binding and the mechanisms of action were not studied in detail (Table 3). For example, a CaM-like protein, CAMLP, in a human pathogen *M. tuberculosis* shown to activate Nicotinamide Adenine Dinucleotide (NAD) kinase and phosphodiesterase (PDE)^61^ plays a role in *M. tuberculosis* growth and survival in macrophages^62,63^. Two CaM-like proteins in *Bacillus subtilis* include CALP, which stimulates PDE and NAD kinase in a Ca^2+^-dependent manner^64^ and YtaF, suggested to activate Ca^2+^ dependent proteins required for sporulation^65^*.* Ca^2+^ binding was quantitatively characterized only in one of the reported bacterial EF-hand proteins, SdrD from *Staphylococcus aureus*^66,67^. This Serine-Aspartate-Repeat (SD repeat) domain protein contains five EF hands within the B1-B5 domains that are responsible for binding Ca^2+^. The binding triggers a conformational change and stabilizes the protein, which enables its interaction with host cells and promotes adhesion^72^. SdrD was also shown to contribute to virulence and inhibition of innate immune response^73^, but mostly through its function as a Sdr family adhesin. To our knowledge, here we present the first detailed report of Ca^2+^-binding properties in a bacterial EF-hand Ca^2+^ sensor, the only EF-hand Ca^2+^ sensor in *P. aeruginosa*.

**Table 3:** Examples of studied and predicted EF hand proteins in bacteria.

Protein name/accession number	Organism	Function	Structural properties	Predicted Subcellular location	Kd for Ca^2+^	References
EfhP AAG07494.1	*P. aeruginosa*	Regulates Ca^2+^ induced virulence and intracellular Ca^2+^ homeostasis	2 EF hands	Periplasmic	13.7 µM	This study,^17^
CasA AF288533	*Rhizobium etli*	Mediates Ca^2+^ dependent symbiosis with leguminous host	3 EF hands	Secreted	NA	^68^
CabD Q9F377_STRCO	*Streptomyces coelicolor*	Affects formation of aerial mycelium	2 EF hands	Cytoplasmic	NA	^69^
CAMLP NP_215727	*M. tuberculosis*	Activates NAD kinase and PDE upon Ca^2+^ binding	1 EF hand	Cytoplasmic	NA	^61,63^
CAMLP AY319523.1	*M. smegmatis*	Activates PDE	1 EF hand	Cytoplasmic	NA	^70^
CALP YP_004243569	*B. subtilis*	Activates PDE in Ca^2+^ dependent manner	NA	Cytoplasmic	NA	^64^
YtfA A0A6I4DFL7	*B. subtilis*	Activates Ca^2+^ dependent proteins required for sporulation	1 EF hand	Membrane	NA	^65^
CLP	*Bordetella pertussis*	Regulates pathogenesis by stimulating adenylate cyclase in Ca^2+^ dependent manner	NA	Secreted	NA	^71^
SdrD SDRD_STAA8	*Staphylococcus aureus*	Promotes adhesion to host surface and resistance	5 EF hands	Anchored to cell wall	16 nM–111 µM	^67,72,73^

Previously, we showed that EfhP mediates Ca^2+^ induction of *P. aeruginosa* virulence in a plant infection model^17^. We predicted a similar outcome for animal model, and here, we showed that deletion of *efhP* decreases the PAO1 survival in macrophages and virulence in *G. mellonella* infection model. This opens a question about specific virulence factors that mediate this impact on *P. aeruginosa* virulence. Our previous studies showed that Ca^2+^ enhances the production of several virulence factors in *P. aeruginosa*, including pyocyanin^16^. Pyocyanin is a redox-active pigment that imposes oxidative stress on airway epithelial cells and mediates tissue damage and necrosis during lung infections^74^. Pyocyanin was also shown to cause death in *G. mellonella* infection model^75,76^. We have previously reported that the deletion of *efhP* completely abolished pyocyanin production in biofilm cells of FRD1, the alginate-producing strain of *P. aeruginosa,* grown at elevated Ca^2+^^17^. In addition, we showed the abundance of several pyocyanin biosynthetic proteins to be significantly reduced in the *efhP* deletion strains in both FRD1 and PAO1 backgrounds^17^. Therefore, we expected that the deletion of *efhP* would have an impact on pyocyanin production in PAO1 grown at elevated Ca^2+^ and used this system to test whether the Ca^2+^ binding ability of EhfP is required for this Ca^2+^-regulation. As predicted, both the *efhP* deletion mutant and its complementation with *efhP_q,* encoding mutated EfhP with abolished Ca^2+^ binding, showed no Ca^2+^ induction of pyocyanin production. This strongly suggests that binding Ca^2+^ enables the role of EfhP in mediating the Ca^2+^ regulation of pyocyanin biosynthesis. Further studies will aim to deduce the exact mechanisms involved in this mediatory role of EfhP.

Overall, a growing body of evidence shows that bacteria possess EF-hand proteins serving as Ca^2+^ sensors which play major roles in the interactions between bacterial pathogens and their hosts. Considering the importance of these proteins and the essential role Ca^2+^ signaling plays in eukaryotes, further studies are necessary to characterize the molecular mechanisms of Ca^2+^ signaling in bacteria. This study presents EfhP as a Ca^2+^ sensor in *P. aeruginosa* that is involved in Ca^2+^ regulation of the pathogen’s virulence. Our current efforts are focused on identifying the protein partners of EfhP and characterizing the cascade of molecular events activated by their interactions which ultimately lead to the regulation of the pathogen’s physiological responses to Ca^2+^.

## Materials and methods

### Bacterial strains and media

The bacterial strains and plasmids used in this study are listed in supplementary Table S1. All the cultures were stored in 10% (v/v) skim milk at − 80 °C. PAO1, the non-mucoid *P. aeruginosa* strain was grown at 37 °C in biofilm minimal medium (BMM)^16^, which contained (per liter): 9.0 mM sodium glutamate, 50 mM glycerol, 0.02 mM MgSO_4_, 0.15 mM NaH_2_PO_4_, 0.34 mM K_2_HPO_4_, and 145 mM NaCl, 200 µl trace metals, 1 ml vitamin solution. Trace metal solution (per liter of 0.83 M HCl): 5.0 g CuSO_4_.5H_2_O, 5.0 g ZnSO_4_.7H_2_O, 5.0 g FeSO_4_.7H_2_O, 2.0 g MnCl_2_.4H_2_O. Vitamins solution (per liter): 0.5 g thiamine, 1 mg biotin (Gold bio). The pH of the medium was adjusted to 7.0. When needed*,* 5 mM CaCl_2_^.^2H_2_O (Sigma) was added. PAO1 was used to obtain genomic DNA for cloning *efhP.* For DNA manipulations, *E. coli* and *P. aeruginosa* cultures were grown in Luria–Bertani (LB) broth (per liter: 10 g tryptone, 5 g yeast extract, 5 g NaCl) at 37 °C with shaking at 200 rpm. Antibiotics used for *E. coli* were (per ml) 50 μg kanamycin (Kan) and 100 μg ampicillin (Amp) and for *P. aeruginosa*, (per ml) 60 μg tetracycline (Tet)*, and* 100 µg carbenicillin (Carb).

*No live vertebrate animals* were used in this study.

### Bioinformatics

The amino acid sequence of EfhP (PA4107) was obtained from the www.pseudomonas.com and submitted to I-TASSER for 3D structure prediction^77^. I-TASSER predicts 3D structure using Protein Data Bank templates and the multiple threading approach LOMETS. PyMol v.1.8.6.0^78^ was used to visualize and highlight the structural features. To test the sequence conservation of *efhP* among *Pseudomonas* species, Basic Local Alignment Search Tools, BLASTN and BLASTP^79^ were used in non-redundant NCBI^80^ and Pseudomonas.com databases^81^. Multiple sequence alignments were performed by using MEGA v.7.0^82^. To study potential correlations between sequence conservation and the isolation sources, an in-house python script was generated by Eric King^83^ and used to extract the isolation source data for each sequence from public databases.

### Cloning, expression, and purification of EfhP

The PAO1 genomic DNA was isolated by using the Wizard Genomic DNA Purification Kit (Promega). A 372 bp region of PA4107 (*efhP*) excluding the 5′ 96 nucleotides encoding the signal peptide was PCR amplified from the *P. aeruginosa* PAO1 genomic DNA using High Fidelity Phusion polymerase (NEB) and primers efhP_om_F and efhP_R, which contain *Nde*I and *Bam*HI recognition sequences (Table S2). The PCR products were subsequently extracted from a 1% agarose gel by Zyppy gel extraction kit (Zymo) followed by a column purification (Zymo). The purified amplicons were digested with *Bam*HI and *Nde*I restriction enzymes and subsequently ligated into the similarly digested pSKB3 vector^84^. Following heat-shock transformation into *E. coli* DH5α^85^, the positive clones were selected on LB supplemented with 50 µg/ml of Kan (Goldbio). Successful transformants were verified by PCR using *efhP*-specific primers (Table S2), restriction analysis using NdeI and BamHI enzymes, and Sanger sequencing at the OSU Sequencing Core Facility using T7 primers (Table S2). The verified construct was named pDAV (Table S1) and heat-shock transformed into *E. coli* Tuner *BL21* (DE3) resulting in EcoREN strain that was used for production of the 6 × His fusion protein. EcoREN was grown at 37 °C in LB supplemented with Kan until the OD_600_ reached 0.6 and induced by the addition of isopropyl β-d-1-thiogalactopyranoside (IPTG) to a final concentration of 1 mM. After 3 h, the cells were harvested by centrifugation at 14,650 g for 30 min and stored at − 20 °C. For lysis, the frozen pellets were thawed on ice, resuspended in 50 mL Buffer A (20 mM Tris, 500 mM NaCl, 10% glycerol, 20 mM Imidazole, pH 7.8). Lysozyme was added to the cell lysate at 0.5 mg/mL. The cells were incubated on ice for 40 min and sonicated in a Fisher ultrasonic processor XL 2010 for a total of 100 s in cycles of 20 s on and 20 s off at power setting of 5 followed by centrifugation at 14,650 g for 30 min at 4 °C. The supernatant was collected and incubated with 4 ml of HisPur Nickel Nitrilotriaceitic acid resin (Ni–NTA) (88,221, Thermofisher), which was pre-equilibrated with buffer A in a 50 ml falcon tube on a rocker for 1 h at 4 °C. The Ni–NTA resin was subsequently transferred into a 25 ml gravity flow column and washed with 100 mL of ice-cold Buffer A. The protein was eluted by using 20 ml buffer B (20 mM Tris, 500 mM NaCl, 10% glycerol, 250 mM Imidazole, pH 7.8). The eluates were collected in fractions of 1.5 ml in microcentrifuge tubes and analyzed by SDS-PAGE to confirm the presence of the purified protein of the correct molecular weight (MW). To cleave the N-terminal 6X His-tag, the eluates containing the protein were pooled and treated with recombinant Tobacco Etch Virus (TEV) protease at 1:40 (TEV: protein sample) molar ratio. The 38 ml mixture was transferred into a dialysis membrane with 12–14 kDa MW cutoff and subjected to dialysis against 1 L buffer (20 mM Tris, 500 mM NaCl, 10% glycerol, pH 8.0) at 4 °C overnight. The non-tagged protein was further purified by a second subtracting Ni–NTA column as described in^86^. Briefly, the dialyzed protein mixture was loaded into a pre-equilibrated Ni–NTA column, and the flow-through was collected as the non-tagged protein. Purified EfhP protein was concentrated by using a spin-column with 10 K MW cut-off (Millipore) to 5–10 mg/ml, aliquoted, and flash frozen in liquid nitrogen for storing at − 80 °C as described in^87^. The mutant EfhP proteins were purified following the same procedure.

Size Exclusion Chromatography (SEC) was carried out using AKTA PURE protein purification system (GE Healthcare Life sciences). For purification purposes, a 120 ml HiLoad column was used, whereas a 24 ml Superdex200 column was used for analytical purposes. Prior to analyses, the columns were equilibrated with 20 mM HEPES, 100 mM NaCl, pH 7.8. The injection volumes on Superdex200 and Hiload columns were 500 µl and 5 ml, respectively. The MWs of the proteins in the eluted fractions were calculated based on the retention volumes and the calibration curve generated by using the Gel Filtration standards (Bio-Rad).

The residual level of Ca^2+^ in the protein samples was determined by using Inductively Coupled Plasma-Optical Emission Spectroscopy (ICP-OES) at the Center of Applied Isotope Studies, University of Georgia, Atlanta. The accurate amount of EfhP in the sample was measured by using amino acid hydrolysis at the Protein Chemistry Laboratory at Texas A and M University. Then the molar ratio of protein to Ca^2+^ was determined. To remove the residual Ca^2+^, the proteins were decalcified by using a Chelex-100 resin (Sigma) column as described in^88^. Briefly, the protein samples were mixed with equal volume of regenerated Chelex-100 resin equilibrated with buffer solution and incubated at 4 °C for 30 min. The remaining Ca^2+^ concentration was measured by BAPTA absorption assay as described in^88^ and was below 0.8 µM.

### Isothermal titration calorimetry (ITC)

ITC measurements were performed by using a Nano-Isothermal Titration Calorimeter III (Calorimetry Sciences Corporation, Utah, USA) or MicroCal PEAQ-ITC (Malvern Instruments Limited, UK). His-tag cleaved protein samples collected from SEC were pooled, dialyzed in HEPES buffer (20 mM HEPES, 100 mM NaCl, pH 7.8), and adjusted to a concentration of 0.1 M. Solutions of CaCl_2_, MgCl_2_, and SrCl_2_ were prepared in the HEPES buffer at 1 mM. Prior to titration, both the protein sample and ligand solutions were filtered through 0.2 µM nylon membrane filter (VWR) by centrifugation for 1 min at 4 ^**0**^C at 15,600 g. A total of 1 ml or 300 µL of purified protein (0.1 M or 50 µM) was loaded into the sample well pre-cleaned with nanopure water using a vacuum pump. Each titration experiment consisted of a total of twenty injections of 10 µl aliquots of ligand solutions with 300 s intervals with Nano ITC III or 18 injections of 2 µl aliquots of ligand solutions with 150 s intervals with MicroCal PEAQ-ITC. After each titration, the isotherm of the decalcified HEPES buffer titrated with the corresponding ligand was subtracted from the protein sample isotherm as the blank. The resulting binding isotherms were fitted by using Bindworks software algorithms followed by modeling for independent binding sites, from which the dissociation constant (Kd) was calculated.

### Fluorescence spectroscopy

To monitor changes in protein surface hydrophobicity, 8-anilino-1-naphthalenesulfonic acid (ANS) (Molecular Probes) was used. In a 1 ml quartz cuvette, 50 µl of 10 µM protein was mixed with 950 µl of 50 µM ANS followed by acquiring an emission spectrum (excitation at 350 nm)^89^ using a Horiba Jobin Yvon (HJY) Fluoromax 3 spectrofluorimeter (JOBIN YVON-SPEX). The effect of Ca^2+^ and Mg^2+^ on EfhP hydrophobicity was determined by titrating protein solution with 1 M CaCl_2_ or MgCl_2_ solutions prepared in HEPES buffer. A total of ten 10 µl aliquots of the ligand solutions were added reaching a final concentration of 10–100 µM. Spectra of HEPES buffer alone and 50 µM ANS in HEPES were collected and subtracted from the EfhP/ANS spectral data, and the differences were plotted at the corresponding ion concentrations. All the measurements were performed three times.

### Nuclear magnetic resonance (NMR)

NMR experiments were performed at 25 °C using a triple resonance ^1^H/^13^C/^15^ N TXI high resolution solution NMR probe in a Bruker DMX 500 MHz spectrometer (www.bruker-biospin.com). The pulse sequence, hsqcetgpsi2 (www.bruker-biospin.com) was optimized and calibrated using the water peak chemical shift, 4.7 ppm as a reference. The ^1^H–^15^ N HSQC experiment with 4092 t_2_ points and 256 t_1_ points was run as described in^90^. The data were processed using nmrPipe^91^ and visualized in NMRFAM-Sparky (T.D. Goddard and D.G. Kneller, SPARKY, University of California, San Francisco). The ^15^ N-labelled 6xHis-tagged proteins were purified as described above and prepared by adding 60 µl of deuterium oxide to 540 µl of 297 µM protein solution. After the initial ^1^H–^15^ N heteronuclear single quantum coherence (HSQC) spectrum was acquired a titration with CaCl_2_ was performed. Aliquots of CaCl_2_ were added to yield final concentrations of 0.03, 0.15, 0.3, 0.6, and 1.5 mM.

### Circular dichroism (CD)

CD experiments were performed on a Jasco J-810 automatic recording spectropolarimeter using 0.05-cm quartz cell cuvette at room temperature. The far-UV (200–250 nm) CD data of Ca^2+^-bound and decalcified protein samples were collected with a protein concentration of 25–100 μM. CD spectra of buffer (15 mM Phosphate or 10 mM HEPES at pH 6.5) at the corresponding Ca^2+^ levels (as was added to the protein sample) were collected as control. 150 mM CaCl_2_ stock solutions were prepared in the same buffers as in protein samples. During data acquisition, the data were averaged over 5 scans with a response time of 4 s and with a scan speed of 50 nm/min. All spectra were corrected by subtracting the blank spectra (buffer containing appropriate amount of Ca^2+^). CD ellipticity values were converted to normalized values (mean molar ellipticity per residue) by standard method according to the manufacturer’s manual. Every experiment was repeated for consistency using freshly purified protein samples.

### Site directed mutagenesis

To study Ca^2+^ binding in EfhP, point mutations were generated and the mutated *efhP* variants were cloned into expression and complementation vectors. The single mutant of EfhP with the D1 (position X) of the 1st EF hand replaced to N was generated by using an inverse PCR with DreamTaq Green PCR Master Mix and primers with introduced point mutation and expression vector pDAV (Table S1, Fig. S1). The template vector was eliminated by DpnI treatment and the product was transformed into heat shock competent DH5α *E. coli*. To generate the double and quadruple point mutations, the 0.52 kb *Xba*I-cleaved fragment of the pMF470 complementation plasmid^17^ containing the full-length *efhP* (468 bp) was cloned into *Xba*I-digested vector pUC19. A series of consecutive point mutations replaced the D1 (position X) and the E12 (position -Z) amino acids of each EF-hand with N and Q, respectively (Fig. S1), using Q5 High-Fidelity DNA Polymerase (New England Biolabs) and primers listed in Table S2. All the mutations were confirmed by Sanger sequencing of the mutated sequences, amplified using *efhP* specific primers (Table S2). The mutated EfhP with single D88N, double D88N/E99Q, D115N/E126Q, and quadruple D88N/E99Q/D115N/E126Q mutations were named for simplicity as EfhP_S, EfhP_D1, EfhP_D2, and EfhP_Q, respectively*.* The corresponding gene portions were amplified from pUC19 cloning vector by using efhP_om_F and efhP_R primers (Table S2) and cloned into pSKB3 as described above. The generated corresponding expression plasmids pBIR102, pBIR104 and pBIR106 were verified by PCR with gene specific primers and by Sanger sequencing using T7 primers (Table S2). These plasmids were transformed into *E. coli* DE3 Tuner cells for protein expression.

For functional studies, the 0.52-kb *Xba*I cleaved fragments of pUC19-derivatives encoding double (D88N/E99Q and D115N/E126Q) or quadruple (D88N/E99Q/D115N/E126Q) mutated full-length *efhP* were cloned into the complementing pMF470. The resulting plasmids, pASK101, pASK102 and pASK106 were introduced into the Δ*efhP* mutant strain PAO1043 by electroporation. The successful transformants were selected by growth on LB agar plates with 300 µg/ml carbenicillin and verified by PCR with gene specific primers (Table S2). The resulting complemented strains were named Δ*efhP::efhP_d1,* Δ*efhP::efhP_d2,* and Δ*efhP::efhP_q* (Fig. S1).

### Pyocyanin extraction

To assess the role of EfhP and its ability to bind Ca^2+^ in the production of pyocyanin, we quantified the production of the pigment in *P. aeruginosa,* PAO1, Δ*efhP,* Δ*efhP*::*efhP* and Δ*efhP::efhP_q* strains by using chloroform-HCl method as described in^92^ with modifications. Cultures were grown in BMM for 18 h and normalized to an OD_600_ of 0.3, following which 100 µl of each culture was plated on BMM plates with or without 5 mM Ca^2+^ and incubated at 37 °C for 24 h. Following incubation, the cells were removed from the agar surface by using a glass spreader and 4 ml of saline and collected in 15 ml falcon tubes. The cell suspensions were briefly vortexed and 500 µl aliquots were collected, pelleted at 15,600 g for 2 min, and stored in − 20 °C for the following quantification of a total protein content by using Bradford method (Alfa Aesar, J61522-AP). The remaining 3.5 ml of cell suspensions were transferred into a 10 ml separatory funnel and mixed with 3.5 ml of chloroform. The mixture was shaken at 200 rpm on a platform at room temperature for 30 min. Following this, the funnels were let to stand in an upright position for 10–15 min until the clear light blue layer was formed at the bottom, which was collected into 20 ml scintillation vials. The chloroform extraction was repeated two more times and the blue extracts were combined, mixed with 3.5 ml of 0.2 N HCl, and shaken at 200 rpm for 30 min. The vials were left to stand in an upright position until a reddish upper layer of pyocyanin was formed. From this layer, 200 µl of the extracts were transferred into a clear-bottom 96-well plate, and absorbance was measured at 520 nm by Synergy Mx 2 Multi-Mode plate reader with Gen5 2.05 PC software (BioTek Instruments). The values were multiplied by an extinction coefficient of 17.1^93^, and pyocyanin production in µg/mg of total cellular protein was calculated.

### Macrophage uptake and killing assays

To evaluate the role of EfhP in *P. aeruginosa* survival during phagocytosis, murine macrophages were infected with wild type PAO1 and Δ*efhP* cultures. J774.1 macrophages (ATCC) were grown in Dulbecco’s modified Eagle’s medium (DMEM; Life Technologies) supplemented with 10% v/v heat-inactivated fetal bovine serum (FBS; Corning Cellgro) and 1% w/v penicillin G sodium salt and streptomycin sulfate (Life Technologies) at 37 °C with 5% CO_2_. For infection assays, cells were seeded at a concentration of 5 × 10^5^ cells/well in 24 well plates in DMEM with 10% FBS. *P. aeruginosa* was grown overnight in lysogeny broth at 37 °C under constant shaking until reaching OD_600_ of 1.0. Bacteria were then washed with phosphate buffered saline (PBS), diluted 1:10 in DMEM supplemented with 10% FBS at a concentration of 5 × 10^8^ CFU/ml, and added to the cells. Plates were centrifuged for 5 min at 1200 rpm and incubated at 37 °C with 5% CO_2_. Cells were incubated with bacteria for 15 min before being washed three times with PBS. To quantify initial adherence and uptake, cells were lysed with distilled water. Cell lysates were then serially diluted and plated on Pseudomonas Isolation Agar (PIA) to quantify viable bacteria. To quantify bacterial survival after initial uptake, cells were incubated with DMEM with 10% FBS and 100 µg/ml gentamycin sulfate (Fisher Scientific) for 90 min to kill extracellular bacteria before being washed three times with PBS and lysed with distilled water. Cell lysates were serially diluted and plated on PIA to quantify viable bacteria. Each experiment contained three technical replicates and was repeated three times on three different days.

### Virulence assay

To assess the role of EfhP in *P. aeruginosa* virulence, *Galleria mellonella* infection model was used as described in^94^ with modifications. This invertebrate model has been established for studying bacterial pathogenesis^76,94^. Active *G. mellonella* larvae (Speedy Worms or American Cricket Ranch) of 2–3 cm length, stored in the dark at 4 °C without feeding for no more than 48 h were chosen for injections. *P. aeruginosa*, PAO1, Δ*efhP,* and Δ*efhP*::*efhP* strains were grown in BMM with 5 mM or no added Ca^2+^ for 12 h (mid log). Cultures were serially diluted to achieve two-five colony forming units (CFU) per injection. To verify the injected infection dose, the remaining cultures prepared for injection were plated on Luria–Bertani (LB) agar plates for CFU count. After the gentle rinse with 70% ethanol followed by 1 mg/mL rifampicin, ten larvae were injected with 5 μL of normalized cell suspension grown at 0 mM or 5 mM Ca^2+^. In addition, five larvae were injected with 5 μL of PBS with or without 5 mM CaCl_2_ to be used as negative controls. The larvae were incubated at 37 °C and tested for death at 24 h, which was selected to exclude the effect of cocooning that begins soon after. Death for each larva was reported when no movement was observed in response to turning over with a sterile toothpick. A total of 50 larvae were used for each experiment, which was repeated five times. The survival rates were averaged between five independent experiments and statistically analyzed by using SPSS (Statistical Package for the Social Sciences) software, IBM, SPSS Statistics for Macintosh, version 28.0.1.0 (142), IBM Corp., Armonk, N.Y., USA.

## Supplementary Information

Supplementary Information.

## Data Availability

The datasets generated or analyzed during the current study are included in this published article and its supplementary information files.
