# Supplementary material for: EF-hand protein, EfhP, specifically binds Ca2+ and mediates Ca2+ regulation of virulence in a human pathogen Pseudomonas aeruginosa
Source: Sci Rep. 2022 May 25;12:8791. doi: 10.1038/s41598-022-12584-9 (PMC9132961; doi:10.1038/s41598-022-12584-9)
Supplement: Supplementary file 1 — Supplementary Information. [file 41598_2022_12584_MOESM1_ESM.pdf]

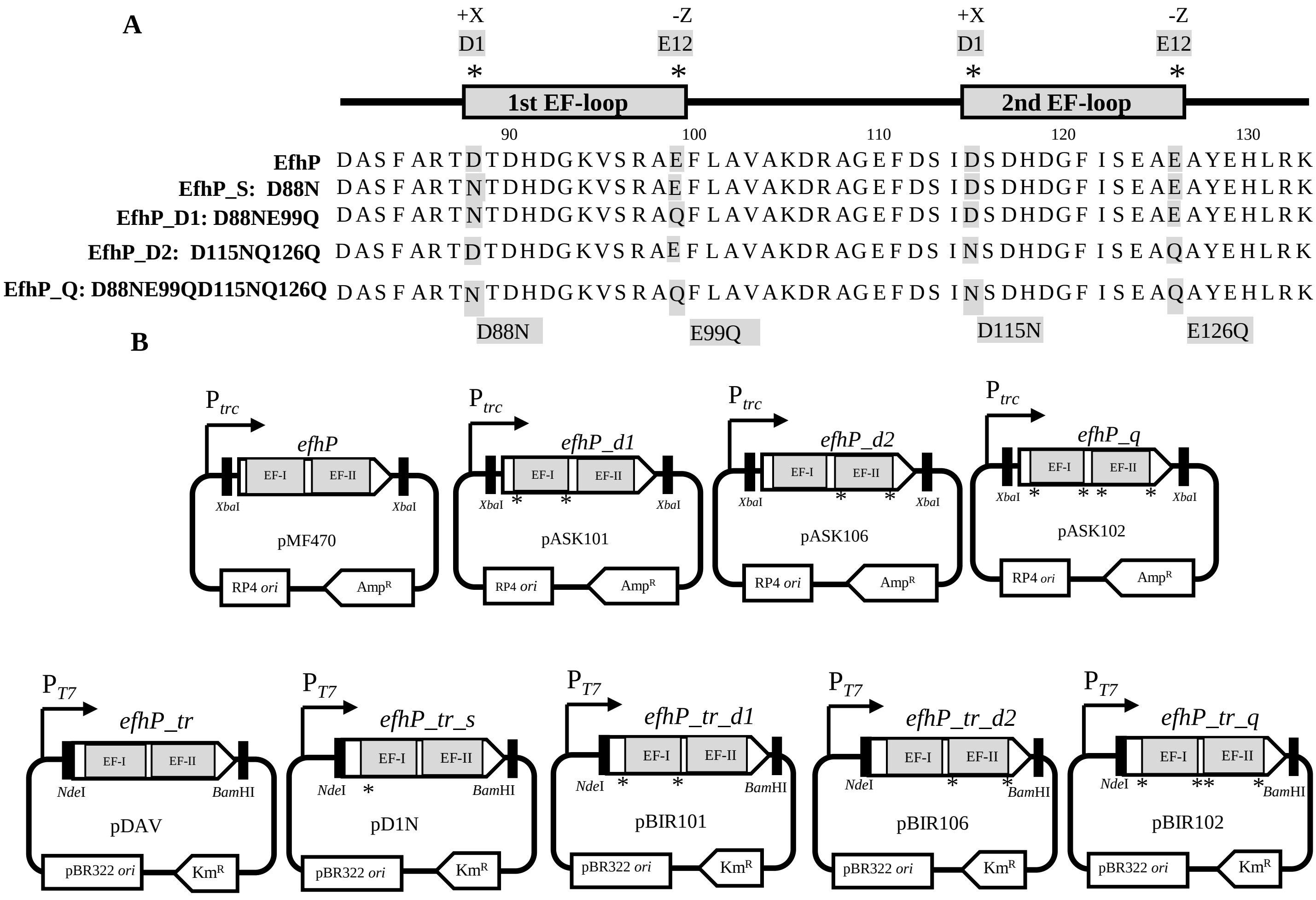

Figure S1. Mutation strategy. (A) Amino acid sequence alignment of EfhP and mutated versions of the protein EfhP_S (EfhP_D88N), EfhP_D1 (EfhP_D88N/E99Q), EfhPD2 (EfhP_D115N/E126Q), and EfhP_Q (EfhP_D88N/E99Q/D115N/E126Q). The D1 (at position +X) and the E12 (at position -Z) of each or both EF hands were replaced to N and Q, respectively to generate double and quadruple mutants. (**B)** Schematics of complementation (top) and expression (bottom) vectors. Full length *efhP* (468 bp) or its mutated versions were cloned into the complementation vector pMF470 for functional studies, whereas *efhP* without the signal peptide (*efhP_tr*) or its mutated versions were cloned into the expression vector pSKB3 for protein purification. Asterisks indicate the mutated residues.

References:

1.Sarkisova, S.A., et al., *A Pseudomonas aeruginosa EF-hand protein, EfhP (PA4107), modulates stress responses and virulence at high calcium concentration.* PLoS One, 2014. **9**(6): p. e98985

2.Deng, J., et al., *Structure of the ROC domain from the Parkinson's disease-associated leucine-rich repeat kinase 2 reveals a dimeric GTPase.* Proceedings of the National Academy of Sciences, 2008. **105**(5): p. 1499-1504.

A.

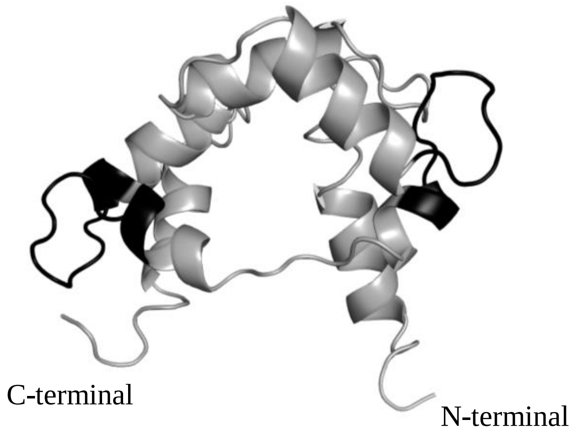

B.

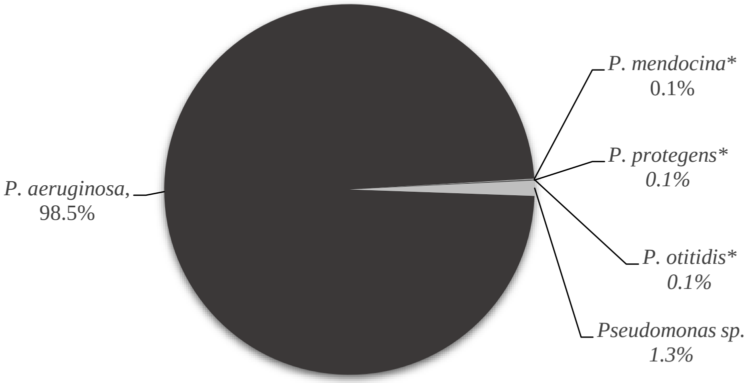

C.

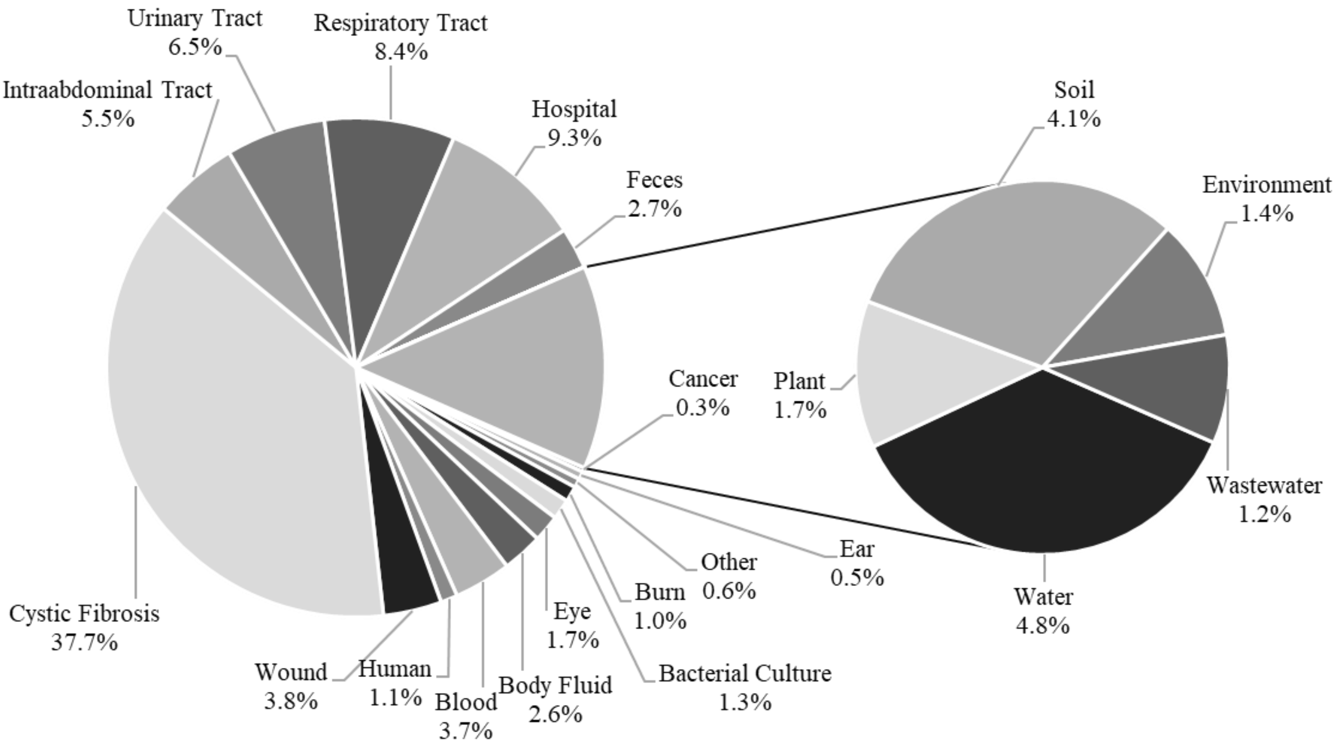

Figure S2. A. I-TASSER 3D predicted model of $EfhP_{tr}$ with truncated signal peptide (1-33 N-terminal residues). EF hand loops are shown in black. The model was visualized by using PyMol v.1.8.6.0. CaM was used as a threading model with the highest Z score of 1.94.
B. Distribution of *Pseudomonas* species encoding *efhP* homologs. The full length *efhP* gene was detected in a total of 1809 strains. The strains of *P. mendocina*, *P. protegens* and *P. otitidis* and 26 *P. sp* strains were identified as *P. aeruginosa* by using average nucleotide identity (ANI) analysis.
C. Distribution of isolation sources for 1809 *P. aeruginosa* strains carrying full-length *efhP* homologs.

A.

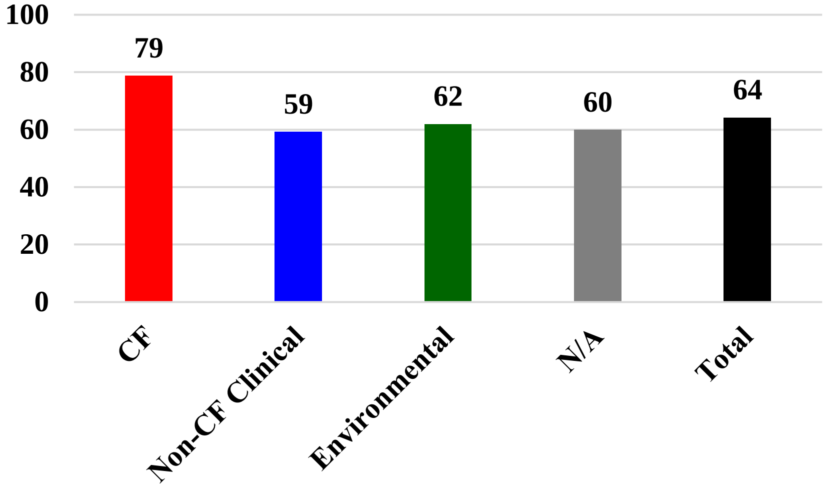

B.

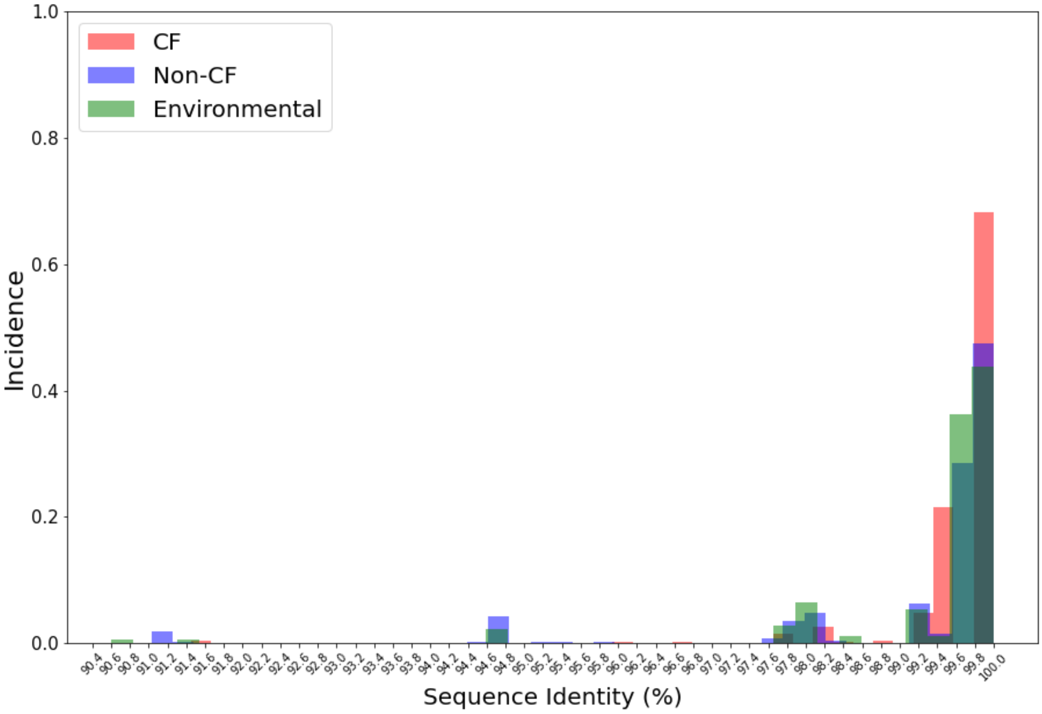

Figure S3. A. Percentage of EfhP-harboring *P. aeruginosa* strains among different groups of isolates: patients with cystic fibrosis (CF), other patients (Non-CF clinical), non-clinical environments (Environmental), or those with no source information (N/A). The total number of all the sequenced and partially sequenced *P. aeruginosa* genomes in the Pseudomonas database at the time of the analyses was 4,643 (Total). **B.** Distribution of incidence related to the conservation of amino acid sequence (sequence identity) among full-length EfhP homologs in *P. aeruginosa* strains isolated from patients with cystic fibrosis (CF), other patients (Non-CF clinical), or from non-clinical environments (Environmental).

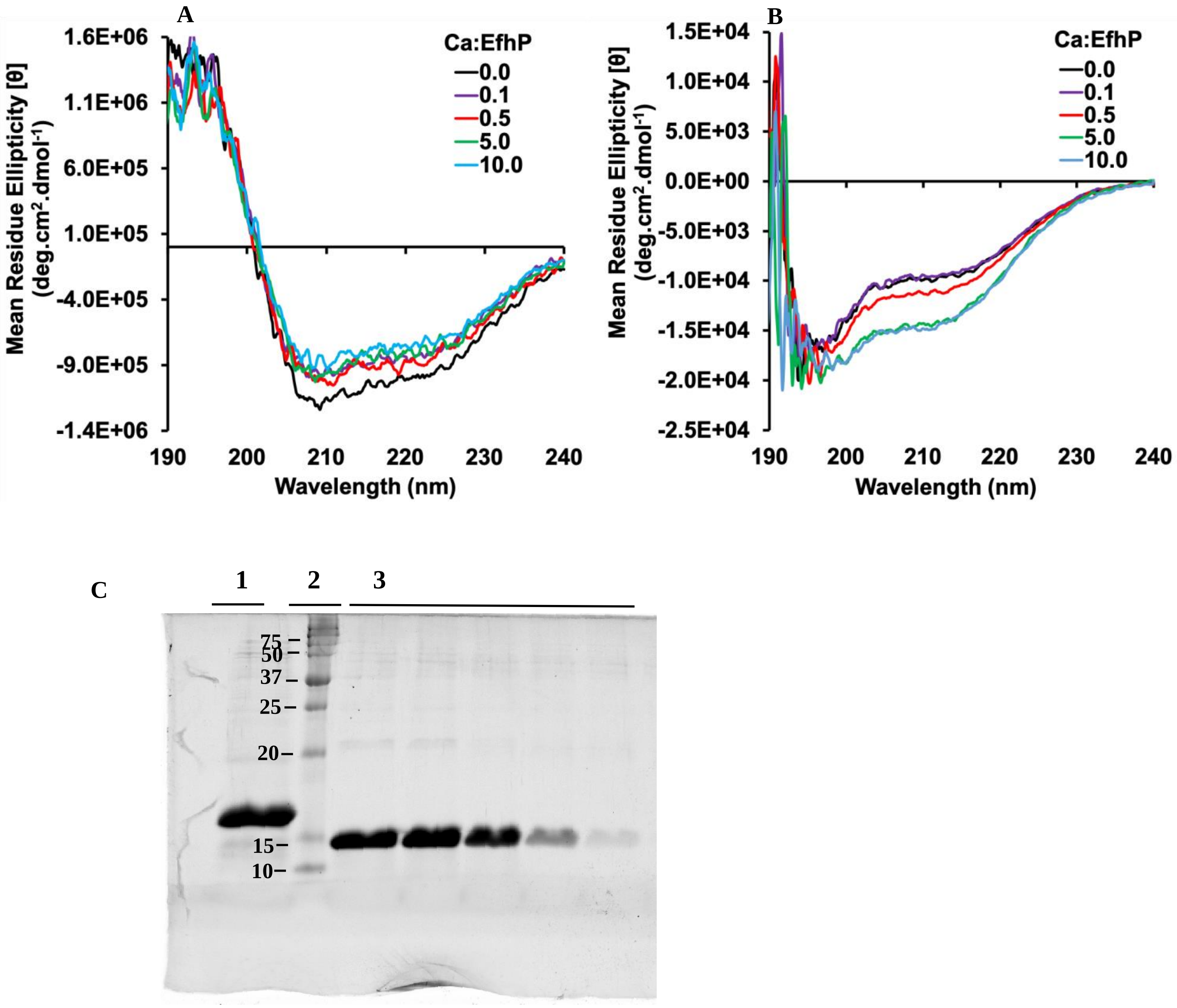

Figure S4. The CD spectra of 25 µM EfhP$_{tr}$ in 15 mM phosphate pH 6.5 buffer (**A**) or 40 µM decalcified EfhP$_{tr}$ in 10 mM HEPES pH 6.5 buffer (**B**) upon titrating with 150 mM $CaCl_2$. (**C**) Coomassie-blue-stained SDS-PAGE gel of the His-Tag-purified EfhP. Lanes: 1. 6X-His-tagged EfhP$_{tr}$ (15.8 kDa), 2. Precision Plus Protein Kaleidoscope Protein Standards (Biorad), 3. His-tag cleaved EfhP$_{tr}$ (12.9 kDa).

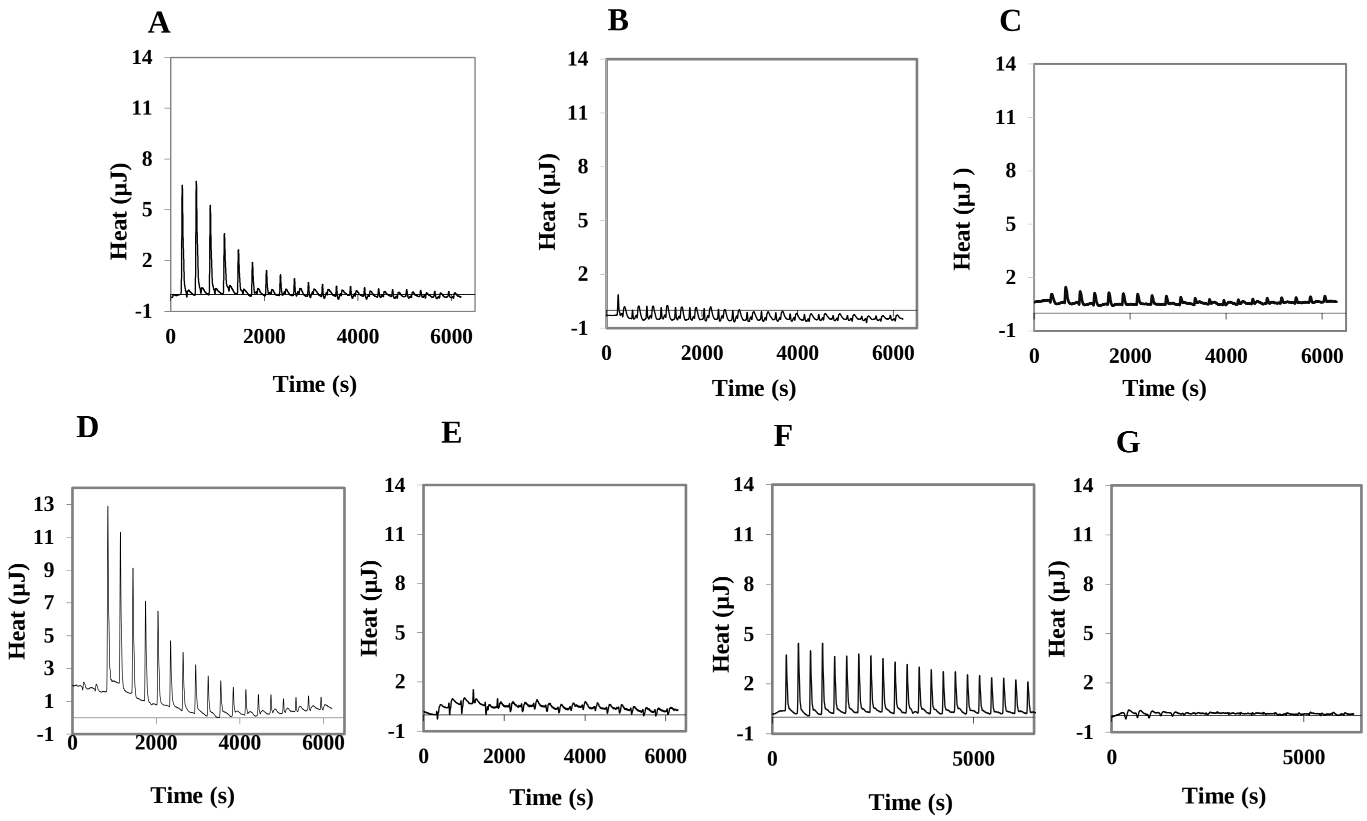

Figure S5. **Representative normalized binding isotherms** for $EfhP_{tr}$ titrated with Ca^{2+} **(A)**, Mg^{2+}**(B)**, and Sr^{2+} **(C)**; EfhP_S **(D),** EfhP_D1 **(E),** EfhP_D2 **(F),** and EfhP_Q **(G)** titrated with Ca^{2+}. 100 µM of proteins in 20 mM HEPES buffer, 100 mM NaCl pH 7.8 was used. The heat signals were detected by using the Nano-Isothermal Titration Calorimeter III. The heat signals of the buffer titrated with the corresponding ions were subtracted as blanks to generate the normalized isotherms.

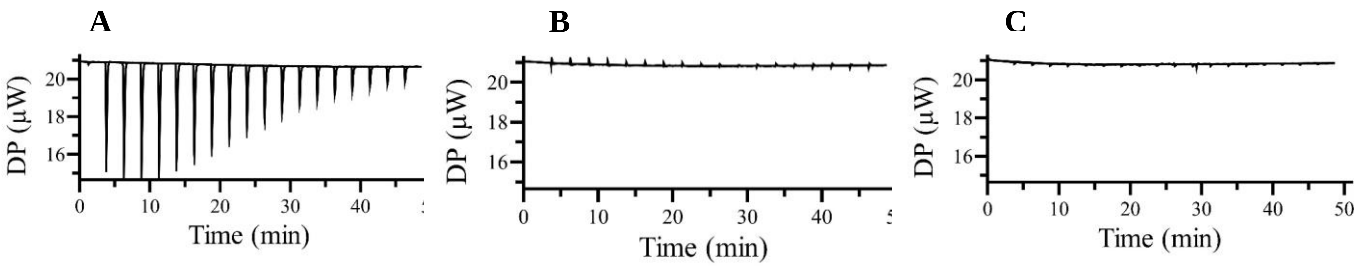

Figure S6. **Representative normalized binding isotherms** for Ca^{2+} -free $EfhP_{tr}$ titrated with Ca^{2+} **(A)**, EfhP_D1 **(B),** EfhP_D2 **(C)**. 50 µM of proteins in 20 mM HEPES buffer, 100 mM NaCl pH 7.8 was used. The heat signals were detected by using the MicroCal PEAQ-ITC (Malvern Instruments Limited, UK). Differential power (DP) in µW is plotted. The heat signals of the buffer titrated with Ca^{2+} were subtracted as blanks to generate the normalized isotherms.

Table S1: List of strains and plasmids used for the study

Escherichia coli		
E. coli DH5α	General purpose cloning strain; Δ(lacZ)M15)	NEB
E. coli BL21 (DE3)	Chemically competent strain for protein expression	Novagen
ECOTEV	DE3 cells for expression of His tagged- Tobacco Etch Virus protease	This study
ECOREN	DE3 cells for the expression of His tagged-EfhP_NTM	This study
ECOBIR2	DE3 cells for the expression of His tagged-EfhP double mutant with the mutation D88NE99Q	This study
ECOBIR6	DE3 cells for the expression of His tagged-EfhP double mutant with the mutation D115NE126Q	This study
ECOBIR4	DE3 cells for the expression of His tagged-EfhP quadruple mutant	This study
Pseudomonas aeruginosa		
PAO1	Wildtype strain, originally isolated from infected burn wound	(1)
pSKB3	pET28b derivative, Kan^r	(2)
pDAV	pSKB3 derivative ,*efhP_ntm,* cloned in NdeI and BamHI sites	This study
pUC19	Cloning vector, Amp^r	NEB
pASK001	pUC19-derivative, 0.52-kb *Xba*I fragment containing *efhP*	This study
pASK002	pASK001-derivative, triplet codon for D88 is replaced with AAC	This study
pASK003	pASK001-derivative, triplet codon for D88 and E99 are replaced with AAC and CAG, respectively	This study
pASK004	pASK001-derivative, triplet codon for D88, E99 and D115 are replaced with AAC, CAG and AAT respectively	This study
pASK005	pASK001-derivative, triplet codon for D88, E99, D115 and E126 are replaced with AAC, CAG, AAT and CAG respectively	This study
pMF470	pMF36, 0.52-kb *Xba*I fragment containing *efhP*	(3)
pD1N	pSKB3 derivative, triplet codon for D88 replaced with AAC	This study
pASK101	pMF470-derivative, triplet codon for D88 and E99 are replaced with AAC and CAG, respectively	This study
pASK102	pMF470-derivative, triplet codon for D88, E99, D115 and E126 are replaced with AAC, CAG, AAT and CAG respectively	This study
pASK106	pMF470-derivative, triplet codon for D115 and E126 are replaced with AAT and CAG respectively	This study
pBIR102	pSKB3 derivative with the double-point mutated EfhPD88NQ99E cloned in *NdeI* and *BamHI* sites of psKB3	This study
pBIR104	pSKB3 derivative with the quadruple-point mutated D88NE99QD115NE126Q cloned *in NdeI* and *BamHI* sites of psKB3	This study
pBIR106	pSKB3 derivative with the double-point mutated D115NE126Q cloned *in NdeI* and *BamHI* sites of psKB3	This study

Table S2: List of strains and plasmids used for the study

**Primer Name**	**Primer Sequences**	**Source**
efhP_F	AGAGAGCATATGGAGCCGCTGGGCCAG	This study
efhP_om_F	AGAGAGCATATGGAGCCGCTGGGCCAG	This study
efhP_R	AGAGAGGGATCCTCAGTGCTGGCCTTG	This study
efhP_D1N_F	AACCAACACCGATCACGATGG C	This study
efhP_D1N_R	GGTGTTGGTTCGGGCAAAGGAG	This study
efhP_E99Q_F	TCTCGCGCGCCCAGTTCCTC	This study
efhP_E99Q_R	ACCGCGAGGAACTGGGCGCG	This study
efhP_D115N_F	TCGACAGCATCAATAGCGAC	This study
efhP_D115N_R	TCATGGTCGCTATTGATGCT	This study
efhP_E126Q_F2	CCCAGGCCTACGAACACCTG	This study
efhP_E126Q_R2	CTGGGCTTCGGAAATGAAGC	This study
T7 Promoter	TAATACGACTCACTATAGGG	(4)
T7 terminator	GCTAGTTATTGCTCAGCGG	(4)

References:

1. Stover, C. K., Pham, X. Q., Erwin, A. L., Mizoguchi, S. D., Warrener, P., Hickey, M. J., Brinkman, F. S., Hufnagle, W. O., Kowalik, D. J., Lagrou, M., Garber, R. L., Goltry, L., Tolentino, E., Westbrock-Wadman, S., Yuan, Y., Brody, L. L., Coulter, S. N., Folger, K. R., Kas, A., Larbig, K., Lim, R., Smith, K., Spencer, D., Wong, G. K., Wu, Z., Paulsen, I. T., Reizer, J., Saier, M. H., Hancock, R. E., Lory, S., and Olson, M. V. (2000) Complete genome sequence of Pseudomonas aeruginosa PA01, an opportunistic pathogen. *Nature* **406**, 959-964

2. Deng, J., Lewis, P. A., Greggio, E., Sluch, E., Beilina, A., and Cookson, M. R. (2008) Structure of the ROC domain from the Parkinson's disease-associated leucine-rich repeat kinase 2 reveals a dimeric GTPase. *Proceedings of the National Academy of Sciences* **105**, 1499-1504

3. Sarkisova, S. A., Lotlikar, S. R., Guragain, M., Kubat, R., Cloud, J., Franklin, M. J., and Patrauchan, M. A. (2014) A Pseudomonas aeruginosa EF-hand protein, EfhP (PA4107), modulates stress responses and virulence at high calcium concentration. *PLoS One* **9**, e98985

4. Aye, S. L., Fujiwara, K., and Doi, N. (2018) A dual system using compartmentalized partnered replication for selection of arsenic-responsive transcriptional regulator. *The Journal of Biochemistry* **164**, 341-348
